# Ischemic and Bleeding Events Associated with Thrombocytopenia and Thrombocytosis after Percutaneous Coronary Intervention in Patients with Acute Myocardial Infarction

**DOI:** 10.3390/jcm9103370

**Published:** 2020-10-21

**Authors:** Ji Woong Roh, Sungmin Lim, Youngdeok Hwang, Kwan Yong Lee, Eun Ho Choo, Ik Jun Choi, Byung-Hee Hwang, Chan Joon Kim, Mahn-Won Park, Dong-Bin Kim, Jong-Min Lee, Chul Soo Park, Hee-Yeol Kim, Ki-Dong Yoo, Doo Soo Jeon, Ho Joong Youn, Wook Sung Chung, Min Chul Kim, Myung Ho Jeong, Youngkeun Ahn, Kiyuk Chang

**Affiliations:** 1 Division of Cardiology, Department of Internal Medicine, Bucheon St. Mary’s Hospital, College of Medicine, The Catholic University of Korea, Seoul 06591, Korea; nomgalda@hanmail.net (J.W.R.); dbkimmd@catholic.ac.kr (D.-B.K.); cumckhy@catholic.ac.kr (H.-Y.K.); 2 Division of Cardiology, Department of Internal Medicine, Yonsei University College of Medicine and Cardiovascular Center, Yongin Severance Hospital, Yongin 17046, Korea; 3 Division of Cardiology, Department of Internal Medicine, Uijeongbu St. Mary’s Hospital, College of Medicine, The Catholic University of Korea, Seoul 06591, Korea; godandsci@catholic.ac.kr (C.J.K.); leejongm@catholic.ac.kr (J.-M.L.); 4 Paul H. Chook Department of information Systems and Statics, Baruch College, CUNY, New York, NY 10010, USA; Youngdeok.Hwang@baruch.cuny.edu; 5 Division of Cardiology, Department of Internal Medicine, Incheon St. Mary’s Hospital, College of Medicine, The Catholic University of Korea, Seoul 06591, Korea; kyle210@naver.com (K.Y.L.); mrfasthand@catholic.ac.kr (I.J.C.); coronary@catholic.ac.kr (D.S.J.); 6 Division of Cardiology, Department of Internal Medicine, Seoul St. Mary’s Hospital, College of Medicine, The Catholic University of Korea, Seoul 06591, Korea; cmcchu@catholic.ac.kr (E.H.C.); hbhmac@catholic.ac.kr (B.-H.H.); younhj@catholic.ac.kr (H.J.Y.); chungws@catholic.ac.kr (W.S.C.); kiyuk@catholic.ac.kr (K.C.); 7 Division of Cardiology, Department of Internal Medicine, Daejeon St. Mary’s Hospital, College of Medicine, The Catholic University of Korea, Seoul 06591, Korea; pmw6193@catholic.ac.kr; 8 Division of Cardiology, Department of Internal Medicine, Yeouido St. Mary’s Hospital, College of Medicine, The Catholic University of Korea, Seoul 06591, Korea; charlie@catholic.ac.kr; 9 Division of Cardiology, Department of Internal Medicine, St. Vincent’s Hospital, College of Medicine, The Catholic University of Korea, Seoul 06591, Korea; yookd@catholic.ac.kr; 10 Division of Cardiology, Department of Internal Medicine, Chonnam National University Hospital, Gwangju 61748, Korea; kmc3242@hanmail.net (M.C.K.); myungho@chollian.net (M.H.J.); cecilyk@hanmail.net (Y.A.)

**Keywords:** acute myocardial infarction, percutaneous coronary intervention, thrombocytopenia, thrombocytosis

## Abstract

The early and late ischemic and bleeding clinical outcomes according to baseline platelet count after percutaneous coronary intervention (PCI) in patients with acute myocardial infarction (AMI) remain unclear. Overall, 10,667 patients from the Cardiovascular Risk and identification of potential high-risk population in AMI (COREA-AMI) I and II registries were classified according to the following universal criteria on baseline platelet counts: (1) moderate to severe thrombocytopenia (platelet < 100 K/μL, *n* = 101), (2) mild thrombocytopenia (platelet = 100~149 K/μL, *n* = 631), (3) normal reference (platelet = 150~450 K/μL, *n* = 9832), and (4) thrombocytosis (platelet > 450 K/μL, *n* = 103). The primary endpoint was the occurrence of major adverse cardiovascular events (MACE). The secondary outcome was Bleeding Academic Research Consortium (BARC) 2, 3, and 5 bleeding. After adjusting for confounders, the moderate to severe thrombocytopenia (HR, 2.03; 95% CI, 1.49–2.78); *p <* 0.001), mild thrombocytopenia (HR, 1.15; 95% CI, 1.01–1.34; *p* = 0.045), and thrombocytosis groups (HR, 1.47; 95% CI, 1.07–2.03; *p* = 0.019) showed higher 5-year MACE rates than the normal reference. In BARC 2, 3, and 5 bleeding outcomes, the bleedings rates were higher than the normal range in the moderate to severe thrombocytopenia (HR, 2.18; 95% CI, 1.36–3.49; *p* = 0.001) and mild thrombocytopenia (HR, 1.41; 95% CI, 1.12–1.78; *p* = 0.004) groups. Patients with AMI had higher 5-year MACE rates after PCI if they had lower- or higher-than-normal platelet counts. Thrombocytopenia revealed higher early and late bleeding rates whereas thrombocytosis showed long-term bleeding trends, although these trends were not statistically significant.

## 1. Introduction

Platelets play an essential role in thrombus formation on erosion or rupture of atherosclerotic plaques, which is the main mechanism of acute myocardial infarction (AMI), and platelets attach to the endothelium, thereby actively participating in atherosclerosis progression [1]. Enhanced platelet function was reportedly associated with systemic inflammation, degree of myocardial damage, and prognosis in patients with AMI [2]. Therefore, abnormal platelet counts may affect clinical outcomes in such patients, and the relationship between platelet counts and clinical outcomes has been evaluated in a few studies.

However, most research has not particularly analyzed bleeding outcomes, and each study used different classifications for platelet count, which do not fit the universal normal range of reference platelet counts (150~450 K/μL) [3]. Moreover, the inclusion criteria of the patients were different, and most studies were only within approximately 1 year. A few studies simultaneously studied both thrombocytopenia and thrombocytosis in patients with AMI [4,5].

Therefore, we analyzed the early and late ischemic and bleeding clinical outcomes in patients classified according to the universal platelet count criteria, including those in patients with thrombocytosis receiving percutaneous coronary intervention (PCI) due to AMI.

## 2. Experimental Section

### 2.1. Study Population

All patients who were diagnosed with AMI and received PCI at index admission between January 2004 and August 2014 were included through the CardiOvascular Risk and idEntificAtion of potential high-risk population in AMI (COREA-AMI) registries I and II, which are multicenter, web-based observational cohort studies. The current registries had updated new clinical and angiographic parameters and evaluated long-term clinical follow-up data collected for as long as possible until 2019. The investigators defined AMI as the criteria for the universal definition of myocardial infarction [6]. AMI was defined by clinical features, including electrocardiographic changes with elevated cardiac biomarkers for myocardial necrosis, and by imaging or pathology. The clinical presentation was classified into two groups: ST-segment elevation myocardial infarction (STEMI) and non-ST segment elevation myocardial infarction (NSTEMI). In our study, only patients with AMI undergoing PCI were enrolled and analyzed. Conservatively managed patients were excluded.

Clinical outcome data were obtained from medical records and through telephone interviews, and angiographic and procedural data were assessed and entered by independent personnel and interventional cardiologists in the participating centers. All the adverse events were determined by source documents and were confirmed centrally by the Committee of the Cardiovascular Center of Seoul, St. Mary’s Hospital, Seoul, Republic of Korea. Mortality was verified from the disqualification of the National Health Insurance Service that is a single government-managed insurance, which covers nearly the entire nation’s population. The study protocol was approved by the Institutional Review Board and was conducted in compliance with the Declaration of Helsinki. This study has been registered on ClinicalTrials.gov (study ID: NCT02806102).

Interventional cardiologists performed coronary angiography and stent implantation according to standard techniques, and the type of the stent was chosen as per the operator’s discretion. Antiplatelet-naïve patients received a loading dose of 300 mg aspirin and 300–600 mg clopidogrel, 60 mg prasugrel, or 180 mg ticagrelor. Additionally, dual antiplatelet therapy (DAPT) with aspirin and P2Y_12_ inhibitors are recommended for all patients for at least 1 year depending on various patient risk factors, given there were no bleeding complications. Using pre-procedural anticoagulant agents such as heparin or low molecular heparin and glycoprotein IIb/IIIa inhibitors and performing aspiration thrombectomy were also at the operator’s discretion. Moreover, optimal medical therapies using statin, beta-blockers, and angiotensin-converting enzyme inhibitors or angiotensin-receptor blockers are recommended for all patients without contraindications. All these therapies were done according to the guidelines.

**Figure 1:**
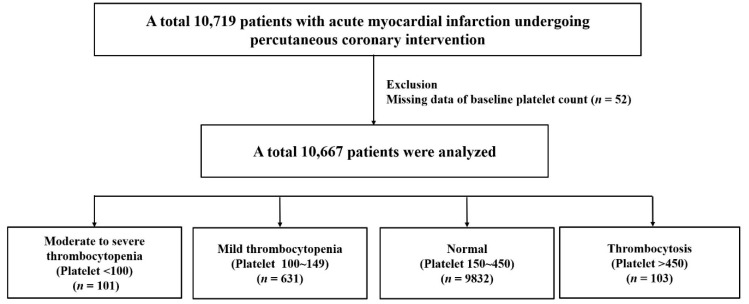
Study flow chart.

Of the 10,719 patients in the AMI cohort excluding those with missing baseline platelet counts (*n* = 52), 10,667 who underwent PCI and received antiplatelet therapy were included in this study (Figure 1). The participants were categorized into 4 universal categorical groups according to the baseline platelet count: moderate to severe thrombocytopenia (platelet < 100 K/μL), mild thrombocytopenia (platelet = 100–149 K/μL), normal reference (platelet = 150–450 K/μL), and thrombocytosis (platelet > 450 K/μL) [3].

### 2.2. Endpoints and Definitions 

The primary endpoint was the occurrence of major adverse cardiovascular events (MACE): a composite of all-cause death, myocardial infarction, and stroke. The secondary endpoint was Bleeding Academic Research Consortium (BARC) 2, 3, and 5 bleeding. Clinical events were defined according to the Academic Research Consortium and American College of Cardiology/American Heart Association clinical trial definition [7,8].

### 2.3. Stastical Analysis

Continuous and categorical variables are reported as mean ± standard deviation and as number and percentage, respectively. Baseline and procedural characteristics among the groups were compared using one-way analysis of variance or Student’s t-test across quartiles of platelet count. Pearson’s χ^2^ test or Fisher’s exact test was used for categorical variables. As a result that differences in baseline characteristics could significantly affect clinical outcomes, adjusted analyses of important confounders were necessary. Multivariate logistic regression analysis models to access the factors associated with thrombocytopenia and thrombocytosis of baseline platelet count were analyzed initially. Any confounding variable factors with *p* value < 0.10 in the univariate analysis were entered into the final multivariate analysis to find factors associated with abnormal platelet count (Supplementary Tables S1 and S2). Thereafter, we could generate adjusted models 1, 2, and 3 using multivariate significant factors in previous logistic regression models to reduce the differences in baseline characteristics. In model 2, we adjusted the significant factors for both thrombocytopenia and thrombocytosis and conventional risk factors for poor outcomes in the AMI population including age, sex, body mass index, diabetes mellitus, hypertension, dyslipidemia, anemia, and left anterior descending artery culprit. Meanwhile in model 3, we adjusted the significant factors for thrombocytopenia or thrombocytosis and conventional risk factors including age, sex, body mass index, diabetes mellitus, hypertension, dyslipidemia, current smoker, previous percutaneous coronary intervention, atrial fibrillation, chronic liver disease, clinical presentation, use of intravenous inotropic agents, left ventricular ejection fraction, anemia, renal insufficiency, left anterior descending artery culprit, and number of treated arteries. Additionally, we performed a multivariate cox analysis with use of the inverse probability weighting method. The multiple logistic regression models estimated the probability weighting. The variables that had a predictive value or significant association with abnormal platelet counts among the baseline characteristics were used for the covariates.

A Kaplan–Meier survival analysis was used to compare the cumulative incidence rates of clinical outcomes, and incidence rates were compared using the log-rank test for early and late outcomes covering a 30-day period. We examined the association between baseline platelet counts as a continuous scale and MACE events or bleeding using the Cox proportional hazard model and cubic spline to assess the expected non-linear relationship. We transformed baseline platelet counts and hazards using a logarithmic scale to stabilize the highly skewed distribution. The log relative hazard was analyzed as the lowest hazard that was observed. Adjusted covariates were similar as the abovementioned variables. All analyses were 2-tailed, and *p* < 0.05 was considered statistically significant. These analyses were performed using SPSS version 23.0 (IBM Corp., Armonk, NY, USA) and R version 3.6.1 (R Foundation for Statistical Computing, Vienna, Austria).

## 3. Results

###  Baseline Characteristics

In 10,667 included patients, the mean age was 63.7 ± 12.8 years, and the male population was 71.4%. There were 101 moderate to severe thrombocytopenia cases, 631 mild thrombocytopenia cases, and 103 thrombocytosis cases upon index admission. The mild thrombocytopenia group had the highest mean age of 68.9 ± 11.9 years and percentage of the male sex (77.8%) among the groups (Table 1). Compared with patients in the normal reference group, patients in the abnormal platelet group were older, had a lower prevalence of being current smokers, and had lower body mass indices. The prevalence of co-morbidities such as diabetes mellitus, hypertension, chronic liver disease, chronic kidney disease, anemia, previous PCI, MI, and stroke was higher in the abnormal platelet groups. At clinical presentation, STEMI was the most common for patients with thrombocytosis, while NSTEMI was the most common for patients with moderate to severe thrombocytopenia. Regarding discharge medications, aspirin and beta-blockers were less prescribed in the abnormal platelet groups excluding the patients who died in the hospital. Angiographic findings showed that the left descending culprit coronary artery had higher prevalence rates in all groups, especially in the thrombocytosis group. The total stent number was high in the normal reference group. The moderate to severe thrombocytopenia group showed longer total stent length with a higher frequency of total stent number ≥ 3 (Table 1).

During the 5-year follow up, of the 2093 (19.6%) patients who died, 1635 (15.3%) died from cardiovascular disease, 466 (4.4%) experienced recurrent myocardial infarction, and 354 (3.3%) experienced stroke events. The clinical outcomes such as the occurrences of MACE, all-cause death, and BARC 2, 3, and 5 bleeding stratified by platelet count are shown in Table 2.

**Figure 2:**
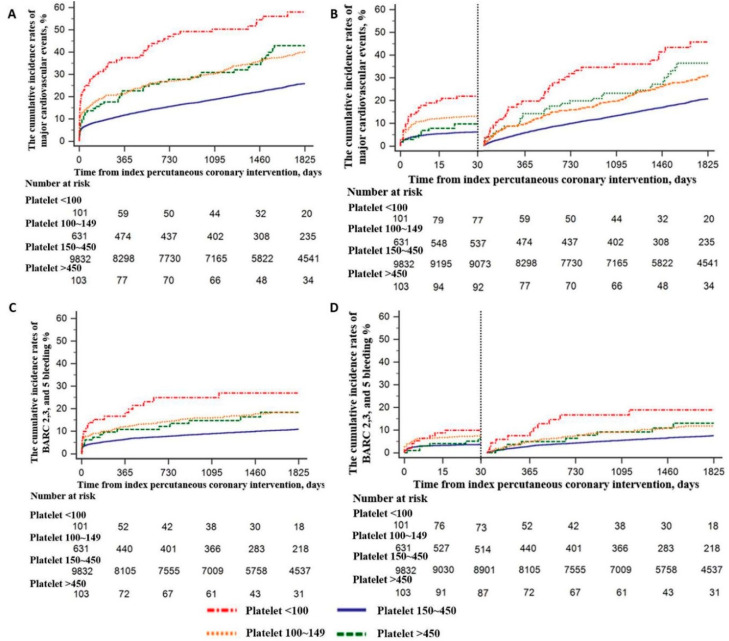
Kaplan–Meier curves for major cardiovascular events (MACE) and Bleeding Academic Research Consortium (BARC) 2, 3, and 5 bleeding according to the baseline platelet counts. (**A**) The 5-year MACE, (**B**) 30-day and 30-day to 5-year MACE, (**C**) 5-year BARC 2, 3, and 5 bleeding, (**D**) 30-day and 30-day to 5-year BARC 2, 3, and 5 bleeding.

The patients in the normal reference group significantly had the lowest rates for MACE, all-cause death, and bleeding both for the 30-day and 5-year outcomes. Kaplan–Meier curves and the log-rank test showed that the proportion of patients with MACE and bleeding was higher in patients in the low and high platelet groups than in the normal reference group (Figure 2).

**Figure 3:**
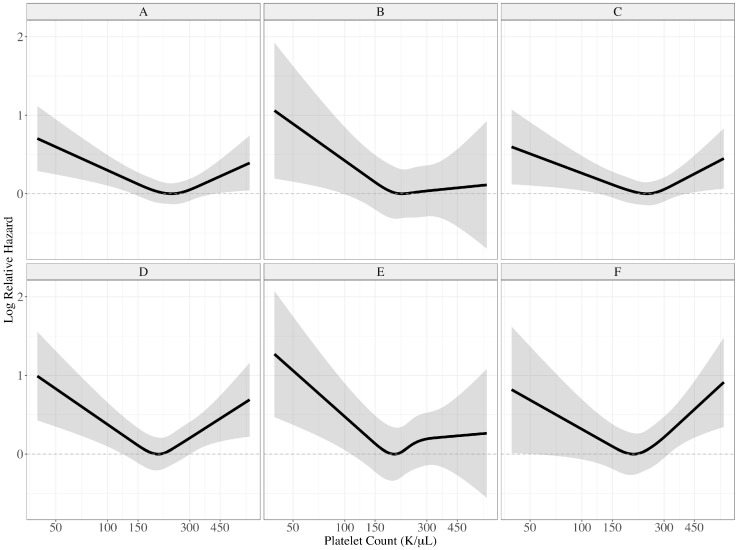
Relative log hazard ratios according to baseline platelet counts for major cardiovascular events (MACE) and Bleeding Academic Research Consortium (BARC) 2, 3, and 5 bleeding. Solid lines represent the calculated log relative hazard, and shaded gray areas represent 95% confidence intervals. (**A**) The 5-year MACE, (**B**) 30-day MACE, (**C**) 30-day to 5-year MACE, (**D**) 5-year BARC 2, 3, and 5 bleeding, (**E**) 30-day BARC 2, 3, and 5 bleeding, (**F**) 30-day to 5-year BARC 2, 3, and 5 bleeding.

Analyzing the association between baseline platelet counts in continuous scales and relative hazard for outcomes, the lowest risk range could be demonstrated, and it could be associated with increased risk of both 5-year MACE and bleeding in both lower and higher platelet counts (*p* for non-linearity <0.001 for 5-year MACE and *p* for non-linearity <0.001 for 5-year bleeding) (Figure 3A,D) The U-shaped relationship in the 30-day to 5-year MACE and bleeding showed a similar pattern to the 5-year outcomes (*p* for non-linearity 0.004 for 30-day to 5-year MACE and *p* for non-linearity 0.001 for 30-day to 5-year bleeding) (Figure 3C,F), whereas it may be a not higher risk of 30-day MACE and bleeding in the higher platelet count group (*p* for non-linearity 0.192 for 30-day MACE and *p* for non-linearity 0.013 for 30-day bleeding) (Figure 3B,E). 

In multivariable adjusted model 3, compared with the normal reference group, the moderate to severe thrombocytopenia (hazard ratio (HR), 2.03; 95% confidence interval (CI), 1.49–2.78); *p <* 0.001), mild thrombocytopenia (HR, 1.15; 95% CI, 1.01–1.34; *p* = 0.045), and thrombocytosis groups (HR, 1.47; 95% CI, 1.07–2.03; *p* = 0.019) had significantly higher 5-year MACE rates. Regarding the 30-day MACE outcomes in the same model, the thrombocytosis group (HR, 1.43; 95% CI, 0.74–2.80; *p* = 0.290) was not significantly different, whereas the moderate to severe thrombocytopenia (HR, 2.22; 95% CI, 1.21–4.10; *p* = 0.010) and mild thrombocytopenia (HR, 1.30; 95% CI 1.03–1.81; *p* = 0.012) groups showed significantly higher MACE rates than the normal reference group. In 30-day to 5-year MACE outcomes, all groups with abnormal platelet counts demonstrated a higher risk of MACE rates (Table 3). 

All-cause death in 30-days, 30-day to 5-year, and 5-year periods presented a similar pattern with MACE. The moderate to severe thrombocytopenia (HR, 2.52; 95% CI, 1.83–3.46; *p* < 0.001), mild thrombocytopenia (HR, 1.31; 95% CI, 1.11–1.54; *p* = 0.002), and thrombocytosis (HR, 1.86; 95% CI, 1.33–2.60; *p* < 0.001) groups all showed significantly higher all-cause death rates than the normal reference group (Supplementary Table S3).

Moreover, 5-year BARC 2, 3, and 5 bleeding were significantly higher in moderate to severe thrombocytopenia (HR, 2.18; 95%CI, 1.36–3.49; *p* = 0.001) and mild thrombocytopenia (HR, 1.41; 95% CI, 1.12–1.78; *p* = 0.004) groups while the thrombocytosis (HR, 1.42; 95% CI, 0.85–2.37; *p* = 0.180) group showed higher bleeding trends without statistical significance (Table 3). The 30-day and 30-day to 5-year BARC 2, 3, and 5 bleeding outcomes showed a similar pattern compared to those of the 5-year bleeding. 

In the analysis using the inverse probability weighting method, the overall results of the risk for 5-year MACE, 5-year BARC 2, 3, and 5 bleeding, and 5-year all-cause death were consistent with the results of the multivariable adjusted models (Supplementary Table S4). One conflicting result was in the 30-day to 5-year MACE that showed borderline statistical significance (HR, 1.10; 95% CI, 0.99–1.30; *p* = 0.052).

## 4. Discussion

The major findings of the present study on the analysis according to the platelet count of AMI patients who received PCI suggest that the (1) 5-year risk of MACE in thrombocytopenia and thrombocytosis groups was significantly higher than in the normal platelet count group. (2) The 5-year risk of BARC 2, 3, and 5 bleeding in the thrombocytopenia group was significantly higher than in the normal platelet group, whereas no statistical significance was seen in the thrombocytosis group. (3) The 30-day risk of MACE and BARC 2, 3, and 5 bleeding was significantly higher in the thrombocytopenia group. (4) The 30-day risk of MACE was not significantly different, whereas the 30-day to 5-year risk of MACE was significantly higher in the thrombocytosis group. The current registry included approximately 8% of patients with abnormal platelet counts, which means 1 of 12 patients in real-world practice. Moreover, most randomized trials for patients undergoing PCI generally did not include patients with abnormal platelet counts according to their exclusion criteria. Therefore, the present study’s results demonstrate clinical significance in these patients.

The measurement of platelet count is an inexpensive and accessible test performed in all hospitalized patients with AMI. A few published studies demonstrated that both thrombocytosis and thrombocytopenia in AMI showed poor short-term clinical outcomes (30 days-1 year) [9,10,11]. Recently, published 2-year AMI data showed a U-shaped association with an increased risk of all-cause mortality in both increased and decreased platelet counts when adjusted for several confounders [12]. We analyzed 5-year data on clinical outcomes including bleeding and divided events into early and late periods. We found that both thrombocytopenia and thrombocytosis were associated with poor long-term clinical outcomes. Furthermore, the early (30 days) and late (30 days-5 years) clinical outcomes on a 30-day basis showed that the thrombocytopenia group had poor MACE outcomes in both periods; however, the thrombocytosis group showed poor MACE outcomes only in the late period. 

Although it is widely recognized that the pre-procedural abnormal platelet count is associated with poor clinical outcomes after PCI in patients with various forms of acute coronary syndrome, adverse outcomes including bleeding during the long-term follow-up has not been previously well-reported. Many studies have recently demonstrated the association between bleeding complications after PCI and adverse clinical outcomes with DAPT [13]. However, analysis on bleeding events according to the platelet count of patients with AMI after PCI has been scarce. One study showed that thrombocytopenia, even a mild one, had a higher risk of bleeding events for 3 years after PCI [14]. Our study also showed worse bleeding outcomes in mild and moderate to severe thrombocytopenia in both early and late periods, whereas there was no significant association between bleeding events and thrombocytosis after adjustment for confounders. Some articles recommended a more potent P2Y_12_ inhibitor after AMI in thrombocytopenia patients [12]. However, because mild and moderate to severe thrombocytopenia also increased the bleeding and ischemic event rates in our research, choosing potent antiplatelet agents should be carefully done. Likewise, for the duration of DAPT, the balancing and trade-off of ischemic and bleeding events should be considered. 

Most previous studies did not categorize platelet count according to the universal criteria [3] as in our research due to a small number of thrombocytosis and moderate to severe thrombocytopenia cases. Recently, a small number of patients with ACS were analyzed retrospectively for long-term mortality according to the universal platelet criteria, wherein thrombocytopenia and thrombocytosis groups had poor clinical outcomes. However, that research only analyzed mortality without bleeding, MI, and stroke events, and thrombocytopenia was not classified into mild and moderate to severe [15]. Although the number of moderate to severe thrombocytopenia and thrombocytosis cases was also small in our research, the 5-year MACE events were significantly higher in both groups with adjusted confounders. As most papers show, the U-shape clinical outcomes according to the baseline platelet counts, the simple evaluation of the platelet count is expected to be a more important factor in predicting the prognosis after PCI of patients with AMI in the future.

Generally, thrombocytosis’ clinical threshold varies per patient as well as its exact definition in the literature, although a platelet count of ≥450 K/μL is the conventionally accepted value in the universal criteria [16]. However, most previous studies could not analyze the universal criteria of thrombocytosis due to a small number of cases. Here, we were able to analyze thrombocytosis in MACE, all-cause death, and bleeding events with adjusting for several confounders, showing poor clinical outcomes of long-term MACE. Additionally, platelets play a role in inflammation in MACE in thrombocytosis patients. Previous studies have indicated that activated platelets can release inflammatory and mitogenic mediators, which would promote more platelet and leukocyte production. Therefore, elevated platelet count at baseline might aggravate inflammation, which can result in more adverse clinical outcomes especially in ischemic cases [11,15]. Interestingly, thrombocytosis also tended to increase bleeding events in our research. A paradoxical risk of bleeding in thrombocytosis has been reported in a few studies, which is explained by various causes such as the von Willebrand factor and platelet abnormal function [17,18]. Therefore, future studies with a larger patient population are needed.

Platelet-dependent thrombus formation is a key factor in AMI pathogenesis. Platelets mediate and initiate thrombotic occlusion of the whole coronary artery and accumulate in the circulation resulting in impaired microcirculation and provoking myocardial ischemia during reperfusion [19]. However, patients with AMI with low platelet counts are commonly older with several comorbidities in most cases [20]. Similarly, our study showed that the patients with low platelet counts were older and had a higher prevalence of diabetes mellitus, hypertension, and previous stroke which could lead to higher rates of ischemic events despite low platelet counts. However, we analyzed the MACE and bleeding events after correcting confounding factors involved in thrombocytosis and thrombocytopenia, and this also showed significantly poor ischemic and bleeding clinical outcomes. Therefore, low and high platelet counts at baseline per se could be used as a short- and long-term simple prognostic markers for AMI after PCI in ischemic MACE including all-cause death or bleeding.

Our study has several limitations. First, because this study was a registry-based analysis, detailed data regarding etiologies of thrombocytopenia or thrombocytosis were lacking. Moreover, we could not evaluate the platelet function, bone marrow or hematologic disease, and chemotherapy or radiation therapy-related abnormal platelet counts. Therefore, the potential mechanism of increased risk of death or bleeding could not be well established. Second, ischemic and bleeding events could be underestimated in a cohort study, even though we tried to gather intensive telephone interviews and chart reviews. Additionally, 30-day mortality rates were approximately 9~20% in the abnormal platelet count groups, and thus early high mortality rates might be related to relatively lower rates of late non-fatal events for long-term follow-up. Third, as our findings were based on a single measurement of pre-procedural platelet level, platelet fluctuations before and after PCI or during the long-term follow-up period were not evaluated, which could have affected clinical outcomes. Lastly, the analysis was divided into the universal criteria of platelet count, and cases of thrombocytosis and moderate to severe thrombocytopenia were relatively few despite the total patient population exceeding 10,000. Therefore, a larger prospective study is necessary in the future.

## 5. Conclusions

Lower and higher platelet counts of patients with AMI both had significantly higher 5-year MACE rates after PCI. Thrombocytopenia revealed significantly higher rates for 30-day early MACE and bleeding and 5-year late bleeding, while thrombocytosis showed higher trends without statistical significance. Therefore, an initial simple assessment of the platelet level is crucial and integrating thrombocytopenia or thrombocytosis in the universal criteria should be considered to assess future long-term ischemic and bleeding events in patients undergoing PCI due to AMI. Further studies are needed for which treatment strategies, such as potency or duration of antiplatelet therapy, are beneficial in patients with abnormal platelet counts.

## Figures and Tables

**Table 1:** Baseline clinical characteristics, discharge medications, angiographic and procedural characteristics according to baseline platelet count.

	Platelet < 100 K/μL (*n* = 101)	Platelet 100~149 K/μL (*n* = 631)	Platelet 150~450 K/μL (*n* = 9832)	Platelet > 450 K/μL (*n* = 103)	*p* Value
**Clinical Characteristics**					
Age, years	68.4 ± 11.2	68.9 ± 11.9	63.2 ± 12.8	67.1 ± 12.4	<0.001
Male	77 (76.2%)	491 (77.8%)	6995 (71.1%)	52 (50.5%)	<0.001
Body mass index, kg/cm^2^	22.7 ± 3.1	23.6 ± 3.4	24.1 ± 3.2	23.1 ± 3.7	<0.001
Diabetes mellitus	39 (38.6%)	247 (39.1%)	3048 (31.0%)	39 (37.9%)	<0.001
Hypertension	54 (53.5%)	369 (58.5%)	5112 (52.0%)	68 (66.0%)	0001
Dyslipidemia	8 (7.9%)	68 (10.8%)	1597 (16.2%)	9 (8.7%)	<0.001
Current smoker	24 (23.8%)	195 (30.9%)	4024 (40.9%)	26 (25.2%)	<0.001
Previous PCI	15 (14.9%)	68 (10.8%)	686 (7.0%)	8 (7.8%)	<0.001
Previous MI	7 (6.9%)	36 (5.7%)	396 (4.0%)	5 (4.9%)	0.099
Previous stroke	12 (11.9%)	63 (10.0%)	689 (7.0%)	12 (11.7%)	0.003
Atrial fibrillation	15(14.9%)	60 (9.5%)	498 (5.1%)	2 (1.9%)	<0.001
Chronic lung disease	4 (4.0%)	18 (2.9%)	232 (2.4%)	4 (3.9%)	0.457
Chronic liver disease	3 (3.0%)	15 (2.4%)	78 (0.8%)	2 (1.9%)	<0.001
Clinical presentation					
STEMI NSTEMI	43 (42.6%)	309 (49.0%)	5394 (54.9%)	62 (60.2%)	0.001
	58 (57.4%)	322 (51.0%)	4438 (45.1%)	41 (39.8%)	0.001
Killip class III~IV	28 (27.7%)	140 (22.2%)	1441 (14.7%)	23 (22.3%)	<0.001
Use of intravenous inotropics	32 (31.7%)	169 (26.8%)	1666 (16.9%)	24 (23.3%)	<0.001
Left ventricular ejection fraction, %	49.9 ± 11.1	51.5 ± 11.2	53.0 ± 10.9	49.8 ± 12.6	<0.001
Left ventricular ejection fraction < 40%	18 (17.8%)	88 (13.9%)	1112 (11.3%)	26 (25.2%)	<0.001
White blood cell count, ×10^3^/μL	9.2 ± 4.8	9.2 ± 5.1	11.6 ± 6.4	13.6 ± 7.2	<0.001
Hemoglobin, mg/L	11.8 ± 2.9	13.0 ± 2.3	13.6 ± 2.1	12.1 ± 2.6	<0.001
Anemia	59 (58.4%)	260 (41.2%)	2834 (28.8%)	52 (50.5%)	<0.001
Serum creatinine, mg/dL	1.7 ± 1.8	1.6 ± 1.6	1.2 ± 1.1	1.2 ± 1.2	<0.001
Estimated glomerular filtration rate, ml/min/1.73 m^2^ *	61.9 ± 32.0	63.6 ± 28.3	76.3 ± 25.5	71.6 ± 27.7	<0.001
Renal insufficiency ^†^	57 (56.4%)	355 (56.3%)	3652 (37.1%)	49 (47.6%)	<0.001
End stage renal disease	10 (9.9%)	36 (5.7%)	187 (1.9%)	2 (1.9%)	<0.001
**Discharge** **medications** ^‡^					
Aspirin	76 (95.0%)	544 (97.3%)	9238 (98.4%)	93 (95.9%)	0.004
P2Y_12_ inhibitor	78 (97.5%)	550 (98.4%)	9318 (99.3%)	97 (100%)	0.021
Statin	70 (87.5%)	492 (88.0%)	8502 (90.6%)	84 (86.6%)	0.090
Beta-blocker	62 (77.5%)	445 (79.6%)	7831 (83.5%)	70 (72.2%)	0.001
ACEI/ARB	60 (75.0%)	437 (78.2%)	7333 (78.1%)	70 (72.2%)	0.485
Anticoagulation	2 (2.5%)	21 (3.8%)	222 (2.4%)	5 (5.2%)	0.067
**Angiographic characteristics**					
Number of coronary arteries involved					0.200
One	38 (37.6%)	257 (40.7%)	4358 (44.3%)	53 (51.5%)	
Two	35 (34.7%)	225 (35.7%)	3244 (33.0%)	26 (25.2%)	
Three	28 (27.7%)	149 (23.6%)	2230 (22.7%)	24 (23.3%)	
Multi vessel disease	63 (62.4%)	374 (59.3%)	5474 (55.7%)	50 (48.5%)	0.079
Culprit coronary artery					<0.001
Left anterior descending artery	46 (45.5%)	260 (41.2%)	4720 (48.0%)	62 (60.2%)	
Right coronary artery	38 (37.6%)	231 (36.6%)	3168 (32.2%)	27 (26.2%)	
Left circumflex artery	15 (14.9%)	103 (16.3%)	1626 (16.5%)	12 (11.7%)	
Left main artery	2 (2.0%)	33 (5.2%)	307 (3.1%)	2 (1.9%)	
**Procedural** **characteristics**					
Treated Coronary artery					
Left anterior descending artery	62 (61.4%)	333 (52.8%)	5919 (60.2%)	73 (70.9%)	<0.001
Right coronary artery	43 (42.6%)	264 (41.8%)	3880 (39.5%)	36 (35.0%)	0.440
Left circumflex artery	25 (24.8%)	165 (26.1%)	2608 (26.5%)	22 (21.4%)	0.664
Left main artery	4 (4.0%)	36 (5.7%)	397 (4.0%)	5 (4.9%)	0.232
Number of treated coronary arteries					0.403
One	71 (70.3%)	490 (77.7%)	7218 (73.4%)	75 (72.8%)	
Two or More	30 (29.7%)	141 (22.3%)	261 4 (26.6%)	2 (27.2%)	
Total stent number	1.5 ± 1.0	1.5 ± 0.8	1.6 ± 0.9	1.5 ± 0.8	0.004
Total stent number ≥ 3	17 (16.8%)	69 (10.9%)	1408 (14.3%)	10 (9.7%)	0.049
Mean stent diameter (mm)	3.1 ± 0.3	3.2 ± 0.4	3.2 ± 0.4	3.2 ± 0.5	0.083
Total stent length (mm)	35.4 ± 24.7	31.9 ± 17.5	34.2 ± 20.5	32.5 ± 19.2	0.035
Thrombus aspiration	9 (8.9%)	93 (14.7%)	1202 (12.2%)	15 (14.6%)	0.170
Use of glycoproteins IIb/IIIa inhibitor	12 (11.9%)	109 (17.3%)	1736 (17.7%)	24 (23.3%)	0.200

Abbreviations: ACEI, angiotensin-converting enzyme inhibitor; ARB, angiotensin receptor blocker; MI, myocardial infarction; NSTEMI: non-ST-segment elevation myocardial infarction, PCI, percutaneous coronary intervention; STEMI, ST-segment elevation myocardial infarction. * Calculated by the chronic kidney disease epidemiology collaboration (CKD-EPI) formula. ^†^ CKD-EPI < 60 mL/min/1.73m^2^.^‡^ Excluded in-hospital death.

**Table 2:** Clinical outcome according to baseline platelet counts.

	Platelet < 100 K/μL (*n* = 101)	Platelet 100~149 K/μL (*n* = 631)	Platelet 150~450 K/μL (*n* = 9832)	Platelet > 450 K/μL (*n* = 103)	*p* Value
**5-year outcomes**					
MACE *	54 (53.5%)	235 (37.2%)	2308 (23.5%)	40 (38.8%)	<0.001
All-cause death	53 (52.5%)	216 (34.2%)	1786 (18.2%)	38 (36.9%)	<0.001
Recurrent myocardial infarction	3 (3.0%)	23 (3.6%)	437 (4.4%)	3 (2.9%)	0.946
Stroke	4 (4.0%)	22 (3.5%)	325 (3.3%)	3 (2.9%)	0.683
Cardiovascular death	33 (32.7%)	161 (25.5%)	1410 (14.3%)	31 (30.1%)	<0.001
BARC 2, 3, and 5 bleeding	20 (19.8%)	96 (15.2%)	929 (9.4%)	15 (14.6%)	<0.001
BARC 3 and 5 bleeding	16 (15.8%)	72 (11.4%)	627 (6.4%)	9 (8.7%)	<0.001
**30-day outcomes**					
MACE *	22 (21.8%)	82 (13.0%)	609 (6.2%)	10 (9.7%)	<0.001
All-cause death	21 (20.8%)	73 (11.6%)	491 (5.0%)	9 (8.7%)	<0.001
Recurrent myocardial infarction	1 (1.0%)	3 (0.5%)	66 (0.7%)	1 (1.0%)	0.892
Stroke	2 (2.0%)	8 (1.3%)	88 (0.9%)	0 (0.0%)	0.268
Cardiovascular death	15 (14.9%)	65 (10.3%)	456 (4.6%)	9 (8.7%)	<0.001
BARC 2, 3, and 5 bleeding	9 (8.9%)	45 (7.1%)	350 (3.6%)	6 (5.8%)	<0.001
BARC 3 and 5 bleeding	8 (7.9%)	41 (6.5%)	320 (3.3%)	5 (4.9%)	<0.001
**30-day to 5-year outcomes**					
MACE *	32 (31.7%)	153 (24.2%)	1699 (17.3%)	30 (29.1%)	<0.001
All-cause death	32 (31.7%)	143 (22.6%)	1295 (13.2%)	29 (28.2%)	<0.001
Recurrent myocardial infarction	2 (2.0%)	20 (3.1%)	371 (3.7%)	2 (1.9%)	0.498
Stroke	2 (2.0%)	14 (2.2%)	237 (2.4%)	3 (2.9%)	0.963
Cardiovascular death	18 (17.8%)	96 (15.2%)	954 (9.7%)	22 (21.4%)	<0.001
BARC 2, 3, and 5 bleeding	11(10.9%)	51 (8.1%)	579 (5.8%)	9 (8.8%)	0.015
BARC 3 and 5 bleeding	8 (7.9%)	31 (4.9%)	307 (3.1%)	4 (4.8%)	0.005

Abbreviations: MACE, major adverse cardiovascular events; BARC, Bleeding Academic Research Consortium. * A composite of all-cause death, myocardial infarction, and stroke.

**Table 3:** Univariate and multivariate hazard ratios according to baseline platelet counts.

	Platelet < 100 K/μL (*n* = 101)		Platelet 100~149 K/μL (*n* = 631)		Platelet 150~450 K/μL (*n* = 9832)	Platelet > 450 K/μL (*n* = 103)	
	HR (95% CI)	*p* Value	HR (95% CI)	*p* Value		HR (95% CI)	*p* Value
5-year MACE							
Univariate	3.17 (2.42–4.16)	<0.001	1.78 (1.55–2.03)	<0.001	Reference	1.83 (1.34–2.50)	<0.001
Model 1 *	2.72 (2.07–3.56)	<0.001	1.35 (1.18–1.55)	<0.001	Reference	1.55 (1.13–2.12)	0.006
Model 2 †	2.43 (1.84–3.20)	<0.001	1.31 (1.14–1.51)	<0.001	Reference	1.49 (1.09–2.04)	0.013
Model 3 ‡	2.03 (1.49–2.78)	<0.001	1.15 (1.01–1.34)	0.045	Reference	1.47 (1.07–2.03)	0.019
5-year BARC 2,3, and 5 bleeding							
Univariate	2.88 (1.85–4.49)	<0.001	1.83 (1.49–2.26)	<0.001	Reference	1.71(1.03–2.84)	0.040
Model 1	2.68 (1.72–4.18)	<0.001	1.66 (1.35–2.06)	<0.001	Reference	1.50 (0.90–2.51)	0.117
Model 2	2.45 (1.57–3.83)	<0.001	1.61 (1.29–2.01)	<0.001	Reference	1.42 (0.85–2.36)	0.184
Model 3	2.18 (1.36–3.49)	0.001	1.41 (1.12–1.78)	0.004	Reference	1.42 (0.85–2.37)	0.180
30-day MACE							
Univariate	3.78 (2.47–5.79)	<0.001	2.18 (1.73–2.74)	<0.001	Reference	1.57 (0.84–2.94)	0.155
Model 1	3.26 (2.13–4.99)	<0.001	1.73 (1.37–2.19)	<0.001	Reference	1.27 (0.68–2.37)	0.459
Model 2	3.19 (2.04–5.02)	<0.001	1.84 (1.43–2.38)	<0.001	Reference	1.39 (0.74–2.61)	0.304
Model 3	2.22 (1.21–4.10)	0.010	1.30 (1.03–1.81)	0.012	Reference	1.43 (0.74–2.80)	0.290
30-day BARC 2,3, and 5 bleeding							
Univariate	2.65 (1.37–5.13)	0.004	2.09 (1.54–2.86)	<0.001	Reference	1.63 (0.73–3.66)	0.233
Model 1	2.47 (1.27–4.79)	0.007	1.92 (1.40–2.63)	<0.001	Reference	1.36 (0.61–3.06)	0.452
Model 2	2.26 (1.16–4.41)	0.016	1.94 (1.41–2.68)	<0.001	Reference	1.31 (0.58–2.95)	0.509
Model 3	1.99 (1.00–4.04)	0.048	1.57 (1.11–2.23)	0.012	Reference	1.22 (0.54–2.74)	0.635
30-day to 5-year MACE							
Univariate	2.95 (2.10–4.15)	<0.001	1.64 (1.40–1.93)	<0.001	Reference	1.95 (1.37–2.79)	<0.001
Model 1	2.55 (1.81–3.58)	<0.001	1.22 (1.04–1.44)	0.016	Reference	1.69 (1.18–2.41)	0.004
Model 2	2.22 (1.58–3.12)	<0.001	1.17 (1.01–1.38)	0.045	Reference	1.55 (1.09–2.22)	0.016
Model 3	1.93 (1.35–2.74)	<0.001	1.11 (1.00–1.32)	0.049	Reference	1.54 (1.07–2.21)	0.020
30-day to 5-year BARC 2,3, and 5 bleeding							
Univariate	2.88 (1.85–4.49)	<0.001	1.83 (1.49–2.26)	<0.001	Reference	1.71 (1.03–2.84)	0.040
Model 1	2.68 (1.72–4.18)	<0.001	1.66 (1.35–2.06)	<0.001	Reference	1.50 (0.90–2.51)	0.117
Model 2	2.45 (1.57–3.83)	<0.001	1.61 (1.29–2.01)	<0.001	Reference	1.42 (0.85–2.36)	0.184
Model 3	2.06 (1.29–3.31)	0.003	1.39 (1.11–1.76)	0.005	Reference	1.45 (0.87–2.43)	0.154

Abbreviations: BARC, Bleeding Academic Research Consortium; CI, confidence interval; HR, hazard ratio; MACE, major adverse cardiovascular events. * Model 1 was adjusted for age and sex. † Model 2 was adjusted for age, sex, body mass index, diabetes mellitus, hypertension, dyslipidemia, anemia, and left anterior descending artery culprit. ‡ Model 3 was adjusted for age, sex, body mass index, diabetes mellitus, hypertension, dyslipidemia, current smoker, previous percutaneous coronary intervention, atrial fibrillation, chronic liver disease, clinical presentation, use of intravenous inotropics, left ventricular ejection fraction, anemia, renal insufficiency, left anterior descending artery culprit, number of treated arteries.

## Supplementary Materials

The following are available online at https://www.mdpi.com/2077-0383/9/10/3370/s1, Table S1: Baseline characteristics associated with thrombocytopenia. Table S2: Baseline characteristics associated with thrombocytosis. Table S3: Univariate and multivariate hazard ratios for all-cause death according to baseline platelet counts. Table S4: Hazard ratios analyzed by inverse probability weighting method according to baseline platelet counts.
