# Supplementary material for: Ischemic and Bleeding Events Associated with Thrombocytopenia and Thrombocytosis after Percutaneous Coronary Intervention in Patients with Acute Myocardial Infarction
Source: J Clin Med. 2020 Oct 21;9(10):3370. doi: 10.3390/jcm9103370 (PMC7589459; doi:10.3390/jcm9103370)
Supplement: Supplementary file 1 [file jcm-09-03370-s001.pdf]

Supplementary Table 1. Baseline characteristics associated with thrombocytopenia.

	Univariate Analysis		Multivariate Analysis	
	HR (95% CI)	*p* Value	HR (95% CI)	*p* Value
Age, years	1.04 (1.03–1.04)	<0.001	1.03 (1.02–1.04)	<0.001
Male	1.42 (1.19–1.70)	<0.001	2.48 (2.04–3.03)	<0.001
Body mass index, kg/cm^2	0.94 (0.91–0.96)	<0.001	0.98 (0.95–1.00)	0.063
Diabetes mellitus	1.43 (1.22–1.66)	<0.001	1.28 (1.09–1.51)	0.003
Hypertension	1.26 (1.08–1.47)	0.003	0.98 (0.83–1.16)	0.840
Dyslipidemia	0.60 (0.47–0.76)	<0.001	0.67 (0.52–0.85)	0.002
Current smoker	0.62 (0.52–0.73)	<0.001	0.73 (0.61–0.88)	<0.001
Previous PCI	1.71 (1.33–2.16)	<0.001	1.33 (0.99–1.77)	0.057
Previous MI	1.49 (1.06–2.03)	0.017	0.91 (0.61–1.34)	0.651
Previous stroke	1.51 (1.16–1.92)	0.001	1.09 (0.83–1.41)	0.531
Atrial fibrillation	2.16 (1.66–2.77)	<0.001	1.48 (1.12–1.92)	0.005
Chronic lung disease	1.28 (0.80–1.94)	0.282		
Chronic liver disease	3.11 (1.80–5.09)	<0.001	3.31 (1.87–5.56)	<0.001
STEMI	0.79 (0.68–0.92)	0.002	0.82 (0.70–0.96)	0.014
Killip class III ~ IV	1.73 (1.44–2.06)	<0.001	1.14 (0.93–1.40)	0.211
Use of intravenous inotropics	1.85 (1.56–2.19)	<0.001	1.43 (1.17–1.75)	<0.001
Left ventricular ejection fraction < 40%	1.31 (1.05–1.62)	0.013	0.867 (0.68–1.08)	0.207
Anemia	1.89 (1.62–2.20)	<0.001	1.26 (1.06–1.50)	0.008
Renal insufficiency†	2.16 (1.85–2.52)	<0.001	1.36 (1.13–1.63)	<0.001
Multi vessel disease	1.19 (1.02–1.38)	0.028	1.09 (0.90–1.31)	0.369
Left anterior descending artery culprit	0.78 (0.67–0.90)	0.001	0.84 (0.71–0.98)	0.031
Left main culprit	1.57 (1.08–2.21)	0.014	1.09 (0.67–1.74)	0.736
Multi vessel treated				
Multi lesion treated	0.83 (0.70–0.99)	0.045	0.81 (0.65–1.01)	0.063
Total stent number ≥ 3	0.80 (0.63–1.00)	0.061	0.87 (0.66–1.13)	0.310
Thrombus aspiration	1.16 (0.93–1.44)	0.177		
Glycoprotein 2b/3a inhibitor	0.92 (0.75–1.12)	0.427		

Abbreviations: MI, myocardial infarction; PCI, percutaneous coronary intervention; STEMI, ST elevation myocardial infarction. †chronic kidney disease epidemiology collaboration (CKD-EPI) <60 ml/min/1.73m^2.

Supplementary Table 2. Baseline characteristics associated with thrombocytosis.

	Univariate Analysis		Multivariate Analysis	
	**HR (95% CI)**	***p* Value**	**HR (95% CI)**	***p* Value**
Age, years	1.02 (1.01–1.04)	0.007	0.99 (0.97–1.01)	0.219
Male	0.40 (0.27–0.60)	<0.001	0.50 (0.32–0.78)	0.002
Body mass index, kg/cm^2	0.91 (0.85–0.97)	0.002	0.94 (0.88–1.00)	0.065
Diabetes mellitus	1.32 (0.88–1.96)	0.172		
Hypertension	1.77 (1.18–2.69)	0.007	1.49 (0.97–2.34)	0.075
Dyslipidemia	0.51 (0.24–0.95)	0.053	0.52 (0.24–0.98)	0.063
Current smoker	0.50 (0.32–0.77)	0.003	0.80 (0.47–1.32)	0.383
Previous PCI	1.07 (0.48–2.08)	0.850		
Previous MI	1.18 (0.41–2.62)	0.724		
Previous stroke	1.69 (0.88–2.98)	0.089	1.35 (0.69–2.42)	0.338
Atrial fibrillation	0.62 (0.06–1.09)	0.137		
Chronic lung disease	1.64 (0.50–3.95)	0.336		
Chronic liver disease	2.16 (0.35–6.95)	0.286		
STEMI	1.19 (0.81–1.77)	0.387		
Killip class III~IV	1.60 (0.98–2.51)	0.048	1.18 (0.70–1.90)	0.516
Use of intravenous inotropics	1.42 (0.88–2.20)	0.139		
Left ventricular ejection fraction < 40%	2.59 (1.62–4.00)	<0.001	1.98 (1.21–3.14)	0.005
Anemia	2.40 (1.62–3.54)	<0.001	1.91 (1.24–2.93)	0.003
Renal insufficiency†	1.59 (1.05–2.37)	0.025	0.89 (0.56–1.41)	0.616
Multi vessel disease	0.74 (0.50–1.10)	0.133		
Left anterior descending artery culprit	1.67 (1.13–2.49)	0.012	1.54 (1.03–2.32)	0.035
Left main culprit	0.59 (0.10–1.88)	0.464		
Multi vessel treated				
Multi lesion treated	1.04 (0.66–1.59)	0.859		
Total Stent number ≥ 3	0.65 (0.32–1.19)	0.202		
Thrombus aspiration	1.21 (0.67–2.03)	0.497		
Glycoprotein 2b/3a inhibitor	1.42 (0.88–2.22)	0.131		

Abbreviations: MI, myocardial infarction; PCI, percutaneous coronary intervention; STEMI, ST elevation myocardial infarction. †chronic kidney disease epidemiology collaboration (CKD-EPI) <60 ml/min/1.73m$^{2.}$

Supplementary Table 3. Univariate and multivariate hazard ratios for all-cause death according to baseline platelet count.

	**Platelet <100 K/μL (*n* = 101)**		**Platelet 100~149K/μL (*n* = 631)**		**Platelet 150~450 K/μL (*n* = 9832)**	**Platelet >450 K/μL (*n* = 103)**	
	**HR (95% CI)**	***p* value**	HR (95% CI)	*p* value		HR (95% CI)	*p*value
5-year all-cause death							
Univariate	3.99 (3.04–5.25)	<0.001	2.13 (1.85–2.45)	<0.001	Reference	2.29 (1.66–3.15)	<0.001
Model 1	3.29 (2.50–4.33)	<0.001	1.53 (1.32–1.76)	<0.001	Reference	1.89 (1.37–2.61)	<0.001
Model 2	2.87 (2.17–3.80)	<0.001	1.50 (1.29–1.74)	<0.001	Reference	1.88 (1.36–2.60)	<0.001
Model 3	2.52 (1.83–3.46)	<0.001	1.31 (1.11–1.54)	0.002	Reference	1.86 (1.33–2.60)	<0.001
30-day all-cause death							
Univariate	4.22 (2.70–6.59)	<0.001	2.40 (1.88–3.07)	<0.001	Reference	1.75 (0.91–3.39)	0.095
Model 1	3.58 (2.28–5.60)	<0.001	1.87 (1.46–2.41)	<0.001	Reference	1.40 (0.72–2.70)	0.320
Model 2	3.45 (2.14–5.56)	<0.001	2.04 (1.55–2.68)	<0.001	Reference	1.58 (0.81–3.06)	0.179
Model 3	2.38 (1.20–4.71)	0.013	1.45 (1.00–2.10)	0.048	Reference	1.73 (0.85–3.53)	0.134
30-day to 5-year all-cause death							
Univariate	3.88 (2.75–5.49)	<0.001	2.01 (1.69–2.40)	<0.001	Reference	2.53 (1.75–3.65)	<0.001
Model 1	3.20 (2.27–4.53)	<0.001	1.40 (1.17–1.67)	<0.001	Reference	2.15 (1.49–3.10)	<0.001
Model 2	2.66 (1.88–3.76)	<0.001	1.34 (1.12–1.61)	0.001	Reference	2.01 (1.39–2.91)	<0.001
Model 3	2.36 (1.64–3.38)	<0.001	1.26 (1.04–1.52)	0.017	Reference	1.99 (1.36–2.90)	<0.001

Abbreviations: CI, confidence interval; HR, hazard ratio. [*]Model 1 was adjusted for age and sex. [†]Model 2 was adjusted for age, sex, body mass index, diabetes mellitus, hypertension, dyslipidemia, anemia, and left anterior descending artery culprit. [‡]Model 3 was adjusted for age, sex, body mass index, diabetes mellitus, hypertension, dyslipidemia, current smoker, previous percutaneous coronary intervention, atrial fibrillation, chronic liver disease, clinical presentation, use of intravenous inotropics, left ventricular ejection fraction, anemia, renal insufficiency, left anterior descending artery culprit, number of treated arteries

Supplementary Table 4. Hazard ratios analyzed by inverse probability weighting methods according to baseline platelet counts.

	**Platelet <100 K/μL (*n* = 101)**		**Platelet 100~149K/μL (*n* = 631)**		**Platelet 150~450 K/μL (*n* =9832)**	**Platelet >450 K/μL (*n* = 103)**	
	**HR (95% CI)**	***p* value**	HR (95% CI)	*p* value		HR (95% CI)	*p* value
5-year MACE	1.88 (1.34–2.63)	<0.001	1.14 (1.01–1.33)	0.048	Reference	1.48 (1.08–2.06)	0.018
5-year BARC 2,3, and 5 bleeding	2.85 (1.67–4.86)	<0.001	1.44 (1.13–1.84)	0.004	Reference	1.12 (0.63–2.02)	0.697
5-year all-cause death	2.46 (1.75–3.45)	<0.001	1.32 (1.12–1.55)	0.001	Reference	1.63 (1.14–2.33)	0.007
30-day MACE	2.05 (1.28–3.29)	0.003	1.36 (1.06–1.75)	0.016	Reference	1.35 (0.74–2.53)	0.413
30-day BARC 2,3, and 5 bleeding	2.17 (1.05–4.49)	0.038	1.48 (1.05–2.09)	0.027	Reference	0.88 (0.35–2.22)	0.777
30-day all-cause death	2.34 (1.42–3.84)	0.001	1.48 (1.14–1.92)	0.003	Reference	0.98 (0.51–1.89)	0.962
30-day to 5-year MACE	1.77 (1.15–2.73)	0.009	1.10 (0.99–1.30)	0.052	Reference	1.59 (1.08–2.23)	0.019
30-day to 5-year BARC 2,3, and 5 bleeding	3.51 (1.66–7.42)	0.001	1.40 (1.00–1.97)	0.049	Reference	1.27 (0.61–2.66)	0.528
30-day to 5-year all-cause death	2.41 (1.57–3.71)	<0.001	1.23 (1.01–1.49)	0.041	Reference	1.95 (1.27–3.00)	0.002

Abbreviations: BARC, bleeding academic research consortium; CI, confidence interval; HR, hazard ratio; MACE, major adverse cardiovascular events. Adjusted for age, sex, body mass index, diabetes mellitus, hypertension, dyslipidemia, current smoker, previous percutaneous coronary intervention, atrial fibrillation, chronic liver disease, clinical presentation, use of intravenous inotropics, left ventricular ejection fraction, anemia, renal insufficiency, left anterior descending artery culprit, number of treated arteries.
